# Supplementary material for: A neuron type-specific microexon in Ank3/ankyrin-G modulates calcium activity and neuronal excitability
Source: bioRxiv. 2025 Dec 16:2025.12.12.693948. Preprint. [Version 2] doi: 10.64898/2025.12.12.693948 (PMC12713133; doi:10.64898/2025.12.12.693948)
Supplement: Supplement 1 — Extended Data Figure 1: ANK3 gene structure and alternative splicing isoforms. The human ANK3 gene, as represented by the UCSC gene models, is divided into multiple segments, with overlaps shown between consecutive segments, to enhance visibility. Constitutive exons are indicated in black and alternative exons are labeled in blue. Not all alternatively spliced exons are shown. Protein domains encoded by specific exons are also indicated on the top of each segment. Extended Data Figure 2: Differential splicing of Ank3 microexon E35a in diverse neuronal cell types. a, Quantification of E35a inclusion in individual glutamatergic and GABAergic neuron types in Tasic 2016 dataset. E35a exon inclusion levels in individual neuronal transcriptional cell types (clusters), grouped by GABAergic and glutamatergic neuronal classes, are shown. b, Similar to (a), but quantification of E35a inclusion in Tasic 2018 dataset. c, Quantification of E35a inclusion in different neuron types in adult mouse cortex and hippocampus. Extended Data Figure 3: Differential splicing of Ank3 microexon E35a in diverse tissues. a, Quantification of E35a inclusion in different adult mouse tissues. b, Quantification of E35a inclusion in different adult human tissues using GTEx RNA-seq data. Extended Data Figure 4: Dynamic Ank3 E35a inclusion in different developmental stages and upon neuronal activation. a, Exon inclusion during the differentiation of mouse embryonic stem cells (mESCs) to glutamatergic excitatory neurons. DIV: days in vitro. Cells at DIV1-28 represent young and maturing neurons. b, Exon inclusion during mouse cortex development. c, Exon inclusion during human cortex development. pcw: post-conception weeks. d, Changes of Ank3 E35a inclusion (left) and Mbnl2 expression (right) in primary hippocampal neurons upon KCl treatment. FDR from differential splicing or expression analysis (derived by the Quantas pipeline) is indicated. The RNA-seq data used for splicing and gene expression quantif [file media-1.pdf]

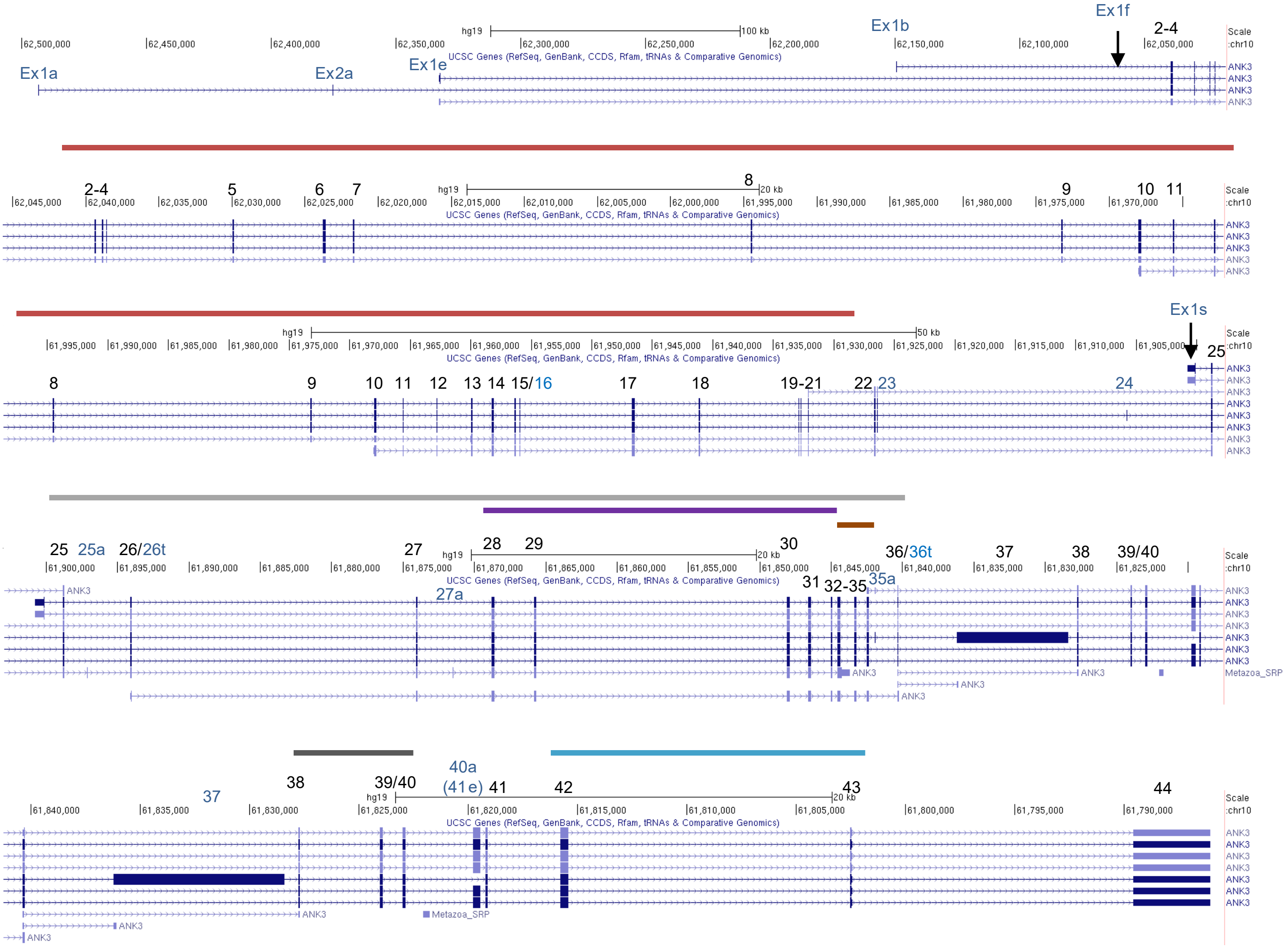

Exon 2-21: Membrane binding domain

Exon 25-36: Spectrin-binding domain
28-32: ZU5N/C
33-35: UPA

38-40: Death domain (DD)

40-43: C-terminal domain (CTD)

Extended Data Fig. 1

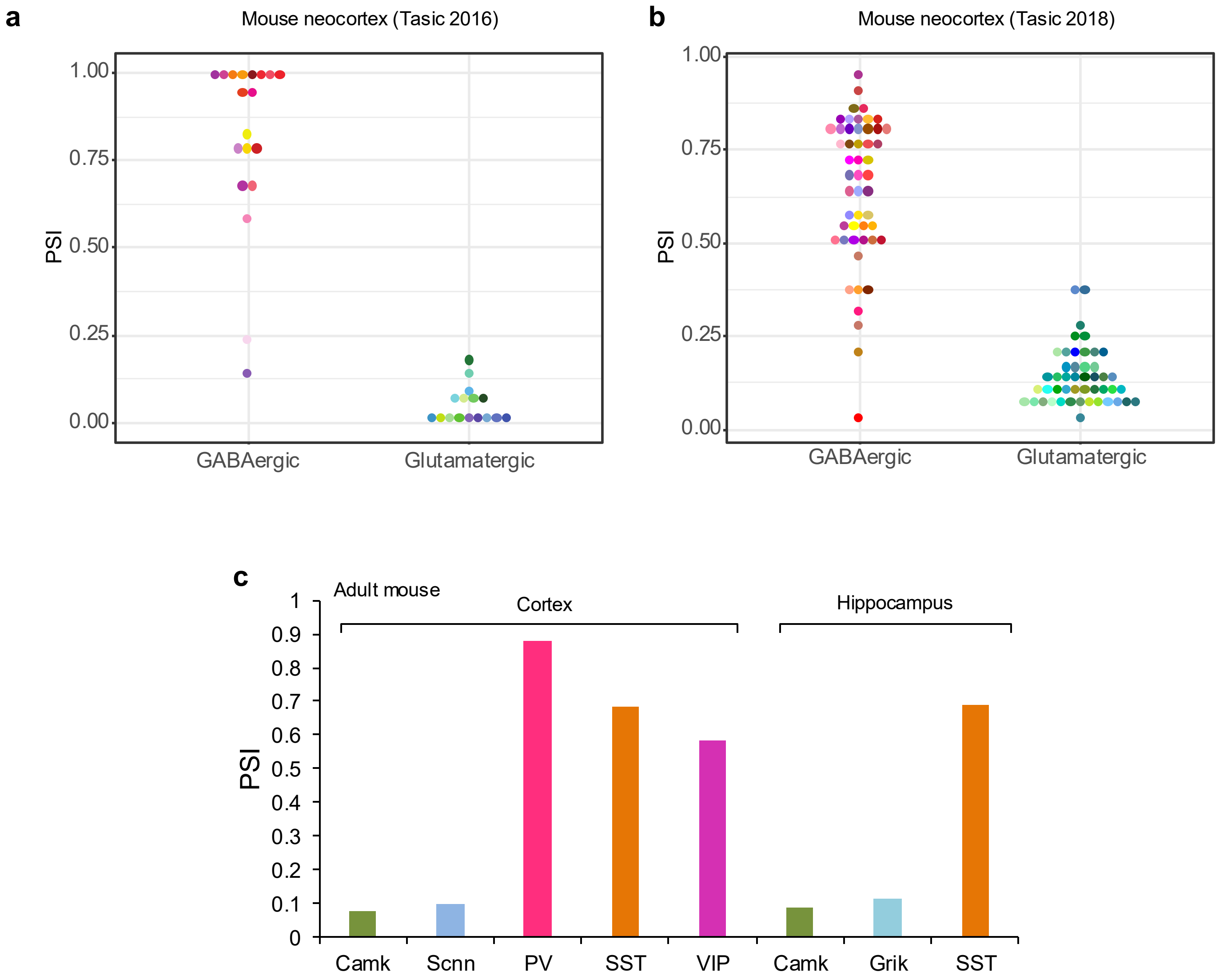

Extended Data Fig. 2

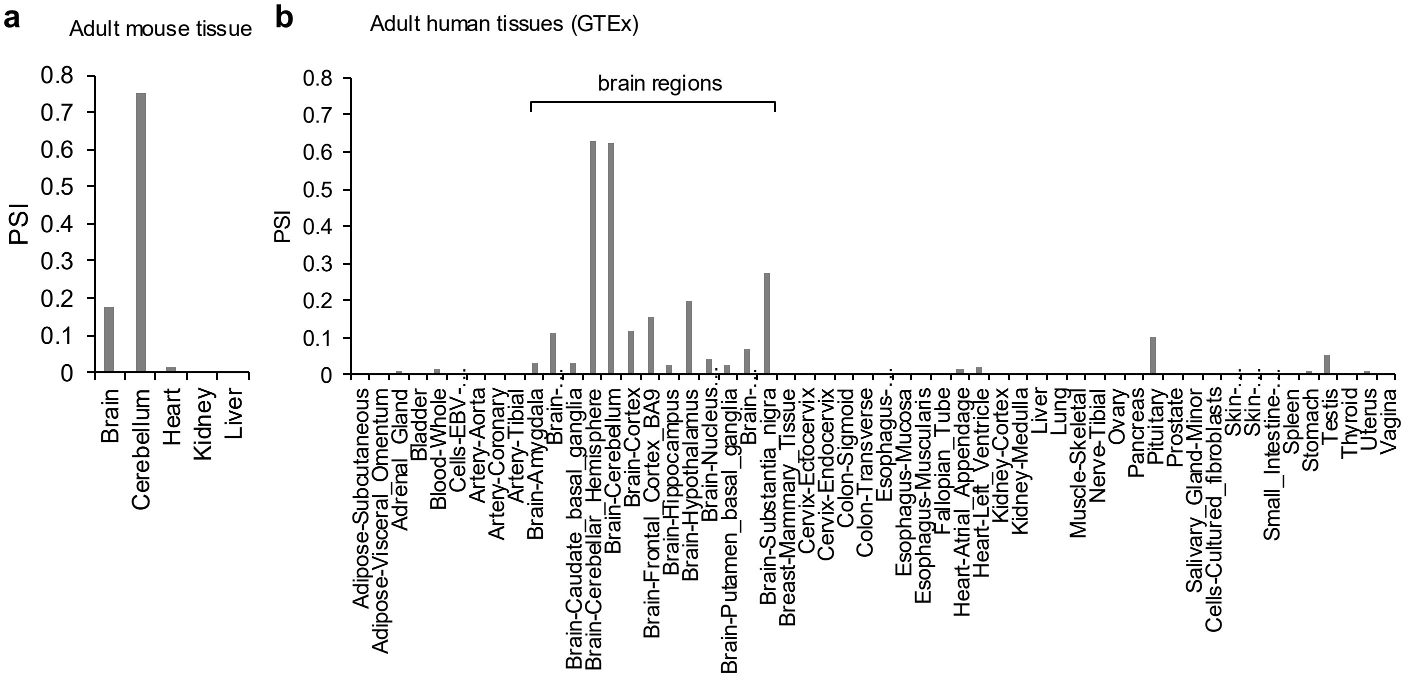

Extended Data Fig. 3

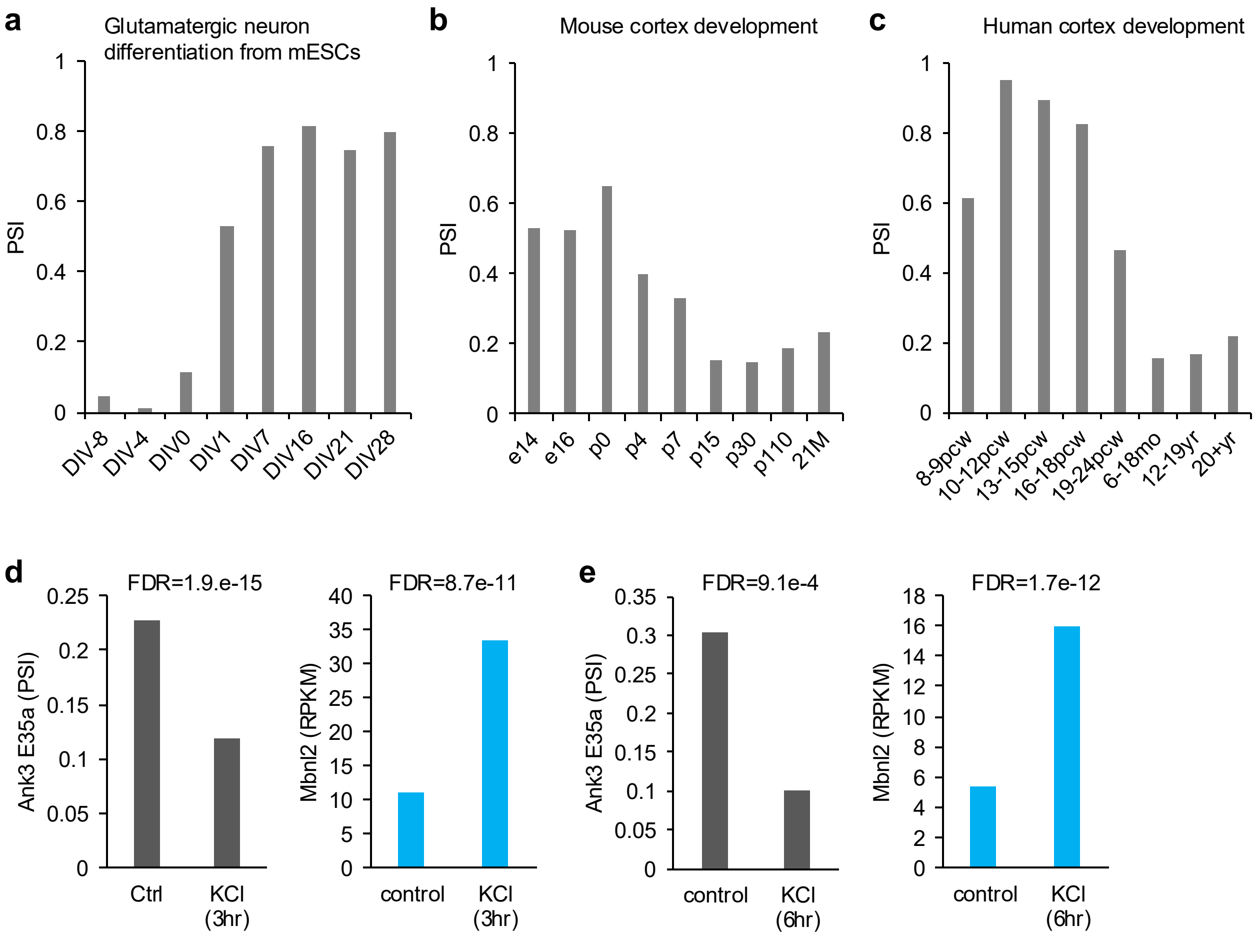

Extended Data Fig. 4

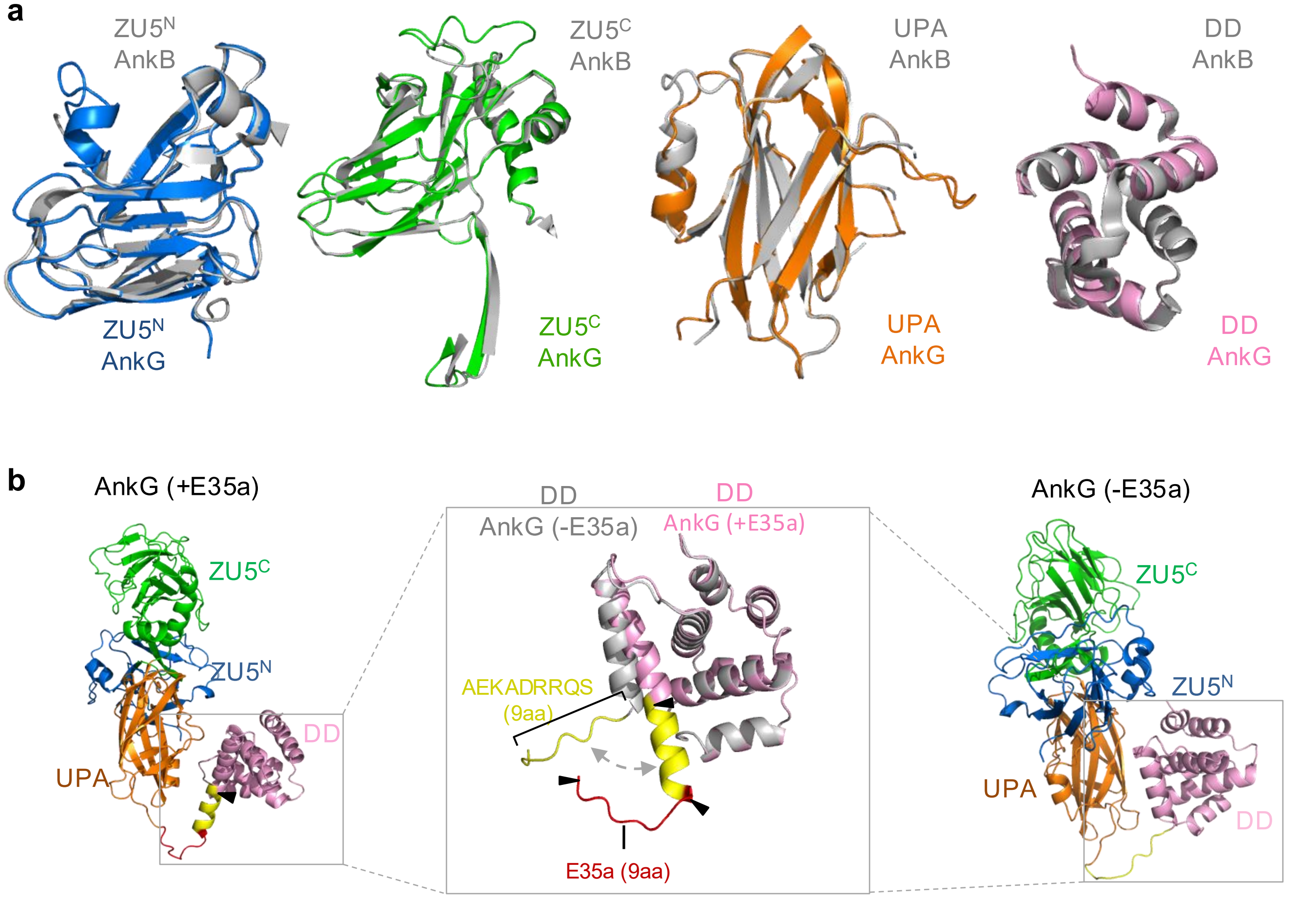

Extended Data Fig. 5

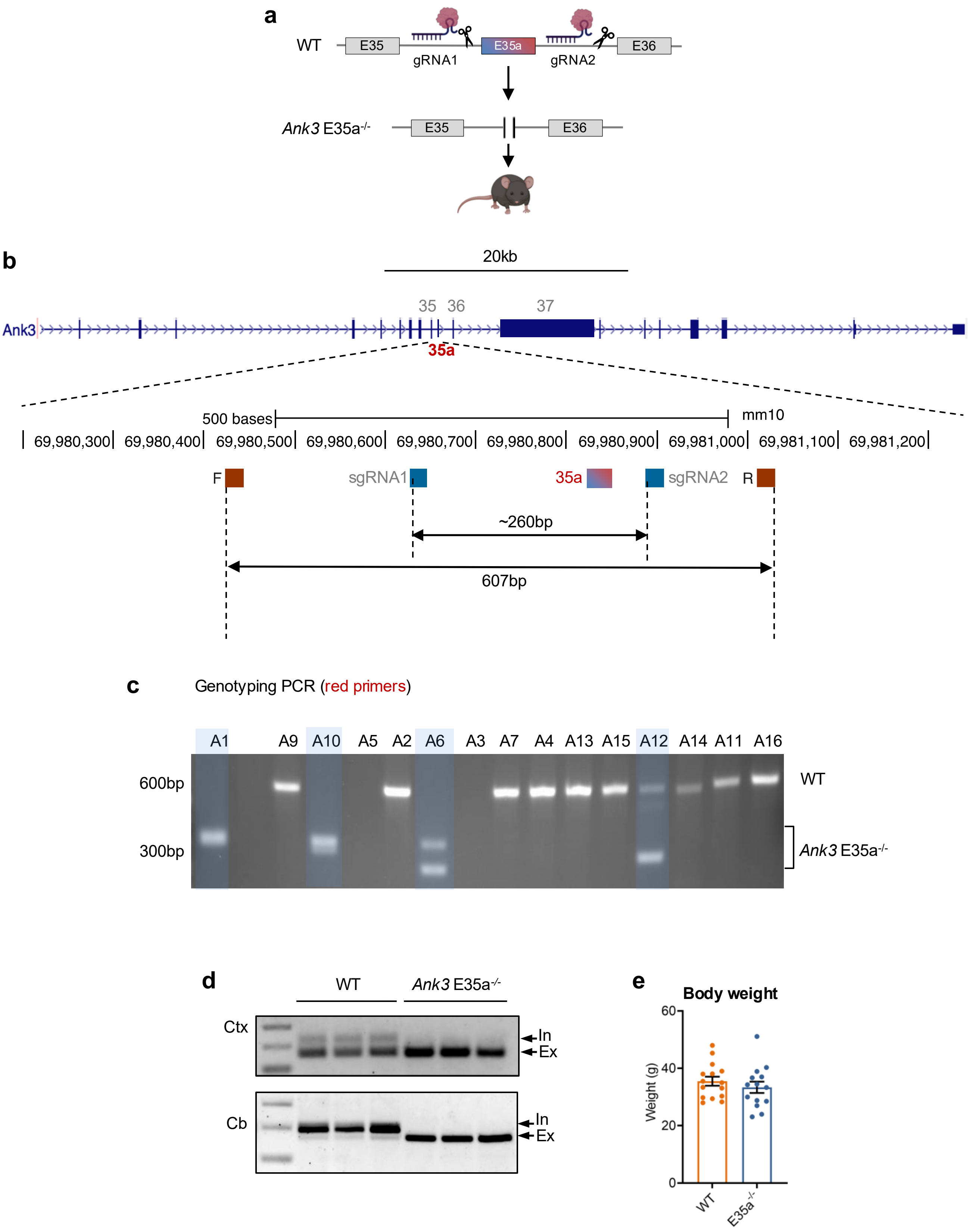

Extended Data Fig. 6

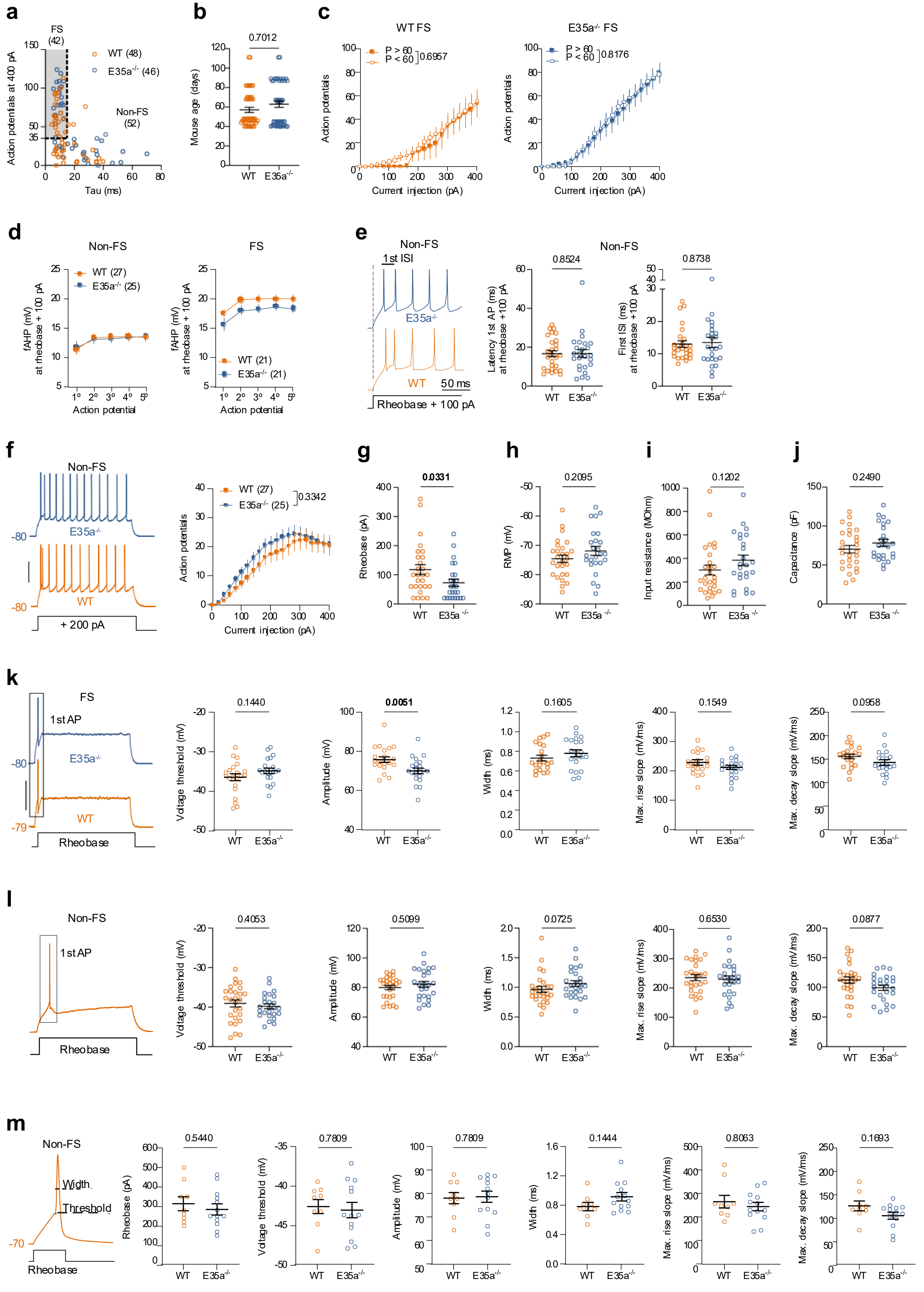

Extended Data Fig. 7

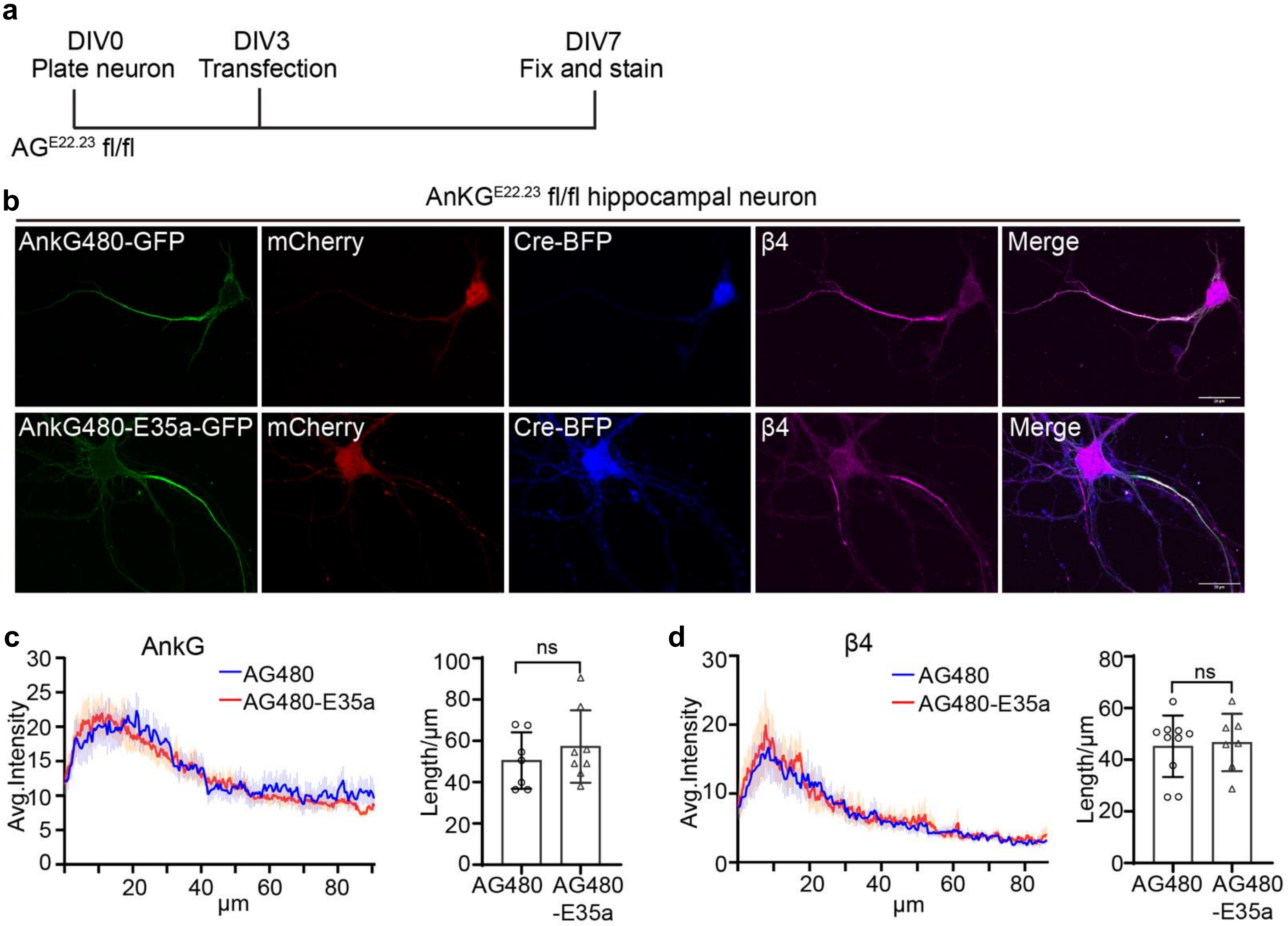

Extended Data Fig. 8

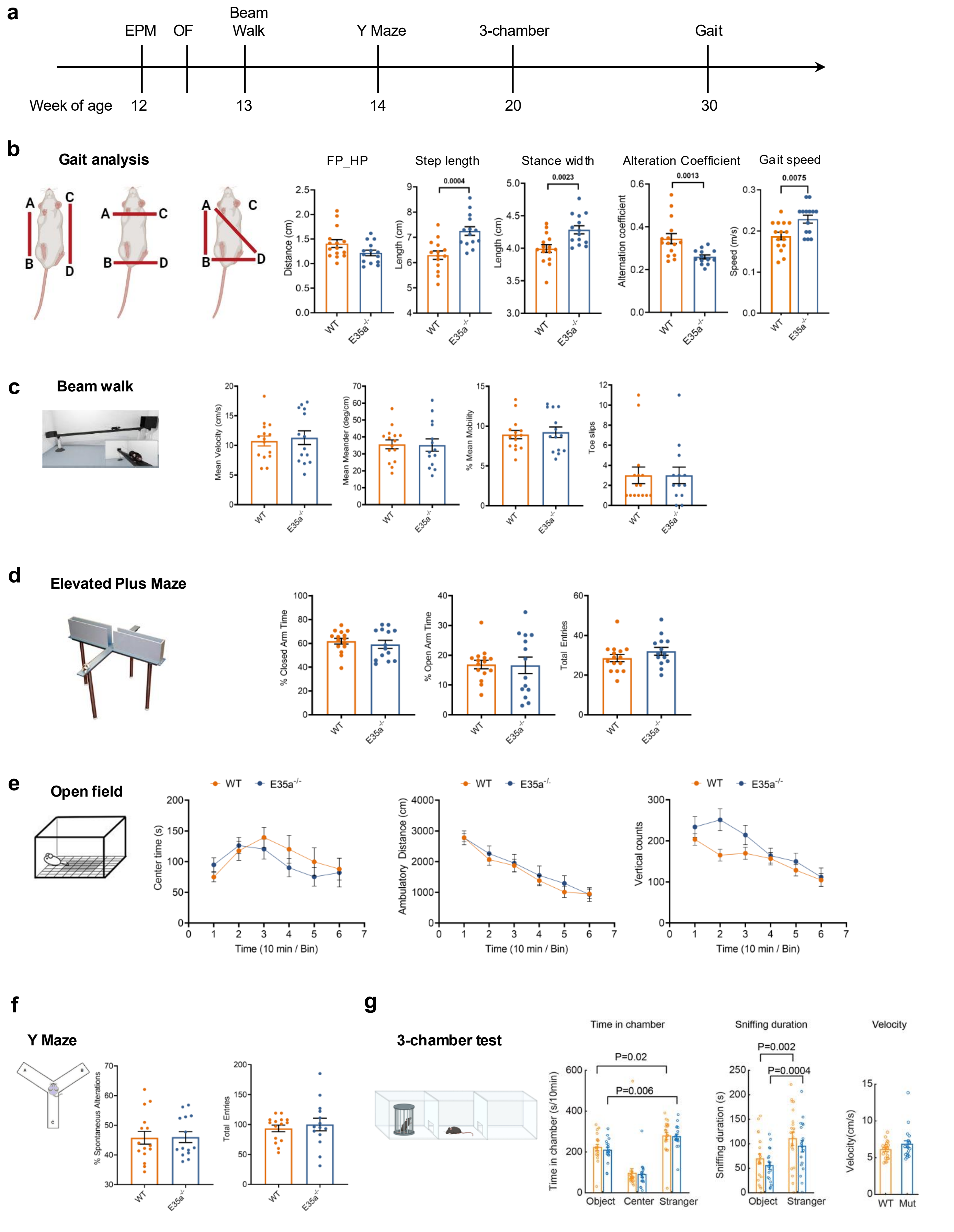

Extended Data Fig. 9
